# Lipomatous Tumors in Pediatric Patients: A Retrospective Analysis of 50 cases

**DOI:** 10.5146/tjpath.2019.01464

**Published:** 2020-01-15

**Authors:** Mine Özşen, Ulviye Yalçınkaya, Zeynep Yazıcı, Mehmet Bartu Sarısözen

**Affiliations:** Department of Pathology, Erzurum Regional Training and Research Hospital, Erzurum, Turkey; Department of Pathology, Uludag University Faculty of Medicine, Bursa, Turkey; Department of Radiology, Uludag University Faculty of Medicine, Bursa, Turkey; Department of Orthopedics and Traumatology, Uludag University Faculty of Medicine, Bursa, Turkey

**Keywords:** Childhood, Lipoma, Lipoblastoma, Liposarcoma

## Abstract

***Objective:*** Although lipomatous tumors are the most common type of mesenchymal tumors in adults, they account for less than 10% of all soft tissue lesions in pediatric patients. In this descriptive study, we aim to present our series of pediatric lipomatous tumors consisting of lipoma, neural fibrolipoma, lipoblastoma, atypical lipomatous tumor, myxoid liposarcoma and pleomorphic liposarcoma, and to evaluate the clinicopathological characteristics of these tumors in reference to the literature.

***Material and Method:*** In this study, pediatric lipomatous tumor cases diagnosed between 2002 and 2018 were screened from pathological archives and retrospectively evaluated.

***Results:*** A total of 50 cases were diagnosed with lipomatous tumor within the mentioned period. Of the total cases, 24 were female (48%) and 26 were male (52%), with age distribution ranging from 1 to 204 months. Histopathological examination revealed lipoma in 26 cases (52%), lipoblastoma in 19 (38%), atypical lipomatous tumor in 2 (4%), myxoid liposarcoma in 2 (4%), and pleomorphic liposarcoma in 1 case (2%).

***Conclusion:*** Although lipomatous tumors are the most common type of mesenchymal tumors; they rarely occur in children. Since there is a limited number of studies on pediatric lipomatous tumors in the literature, there is insufficient data on the prevalence and incidence of these tumors. These tumors may slowly enlarge to greater sizes, especially those localized in deep tissues, and may cause various clinical symptoms by compressing surrounding tissues. Local recurrences may occur, even after total excision, and require close monitoring.

## INTRODUCTION

Lipomatous tumors including lipoma, lipoblastoma, hibernoma, atypical lipomatous tumor and liposarcoma play an important role in soft tissue pathology, as they are common in adults. Lipoma, the most common soft tissue tumor, accounts for 16% of all adult soft tissue tumors. Although lipomatosis can be detected from 2 years of age, patients are usually diagnosed in adulthood. The average diagnosis age for hibernoma is 38 years, and only 5% of patients are diagnosed under the age of 18. Unlike other types of benign lipomatous tumors, 90% of patients with lipoblastoma are diagnosed under 3 years of age, and very few are adolescents or adults. Atypical malignant lipomatous tumors, which account for 40-45% of all liposarcomas, are rarely seen in children. Myxoid liposarcoma, accounting for 15-20% of liposarcomas and 5% of soft-tissue sarcomas, typically occurs in adulthood, and is the most common type of liposarcoma in children and adolescents (1–6). Lipomatous tumors account for less than 10% of soft-tissue lesions within the first two decades of life (7).

In this descriptive study, we aim to present our series of pediatric lipomatous tumors including lipoma, neural fibrolipoma, lipoblastoma, atypical lipomatous tumor, myxoid liposarcoma and pleomorphic liposarcoma, and evaluate the clinicopathological characteristics of these tumors in reference to the literature.

## MATERIAL and METHODS

In this study, we screened the archives of Faculty of Medicine, Department of Medical Pathology for the pediatric lipomatous tumor cases diagnosed between 2002 and 2018. The hematoxylin and eosin-stained slides were re-examined, and histopathological features were documented. The cases were evaluated retrospectively according to age, gender, localization, and clinicopathological features. The study was approved by the local Clinical Research Ethics Committee, dated 17 September 2018 and numbered 2018-15/3.

## RESULTS

A total of 50 cases were diagnosed with lipomatous tumors within the mentioned period (Table 1). There were 24 females (48%) and 26 males (52%), with an age distribution ranging from 1 to 204 months (mean value of 73+63.3 months). The histopathological examination revealed lipoma in 26 cases (52%), lipoblastoma in 19 (38%), atypical lipomatous tumor in 2 (4%), myxoid liposarcoma in 2 (4%), and pleomorphic liposarcoma in 1 case (2%).

**Table 1:** Clinicopathological findings of cases (n=50).

**Variable**		**No. of cases (%)**
Gender	Female Male	24 (48) 26 (52)
Age	≤4 4-10 >10	24 (48) 13 (26) 13 (26)
Type	Lipoma Fibrolipoma Intramuscular lipoma Spindle cell lipoma Lipoblastoma Atypical lipomatous tumor Pleomorphic liposarcoma Myxoid liposarcoma	26 (52) 3 (6) 2 (4) 1 (2) 19 (38) 2 (4) 1 (2) 2 (4)
Tumor site	Head and neck Lower extremity Lumbosacral Back Upper extremity Gluteus Inguinal Intradural-extramedullary Others	9 (18) 10 (20) 7 (14) 5 (10) 4 (8) 3 (6) 2 (4) 2 (4) 8 (16)

Of the 26 cases diagnosed with lipoma, 15 were male (57.7%) and 11 were female (42.3%). The mean age of the cases was 97.3+56.2 months (range:4-204 months). The mean tumor diameter was 4.84+3.1 cm (range:1.1-12.5 cm).

Locations of lipomatous tumors were as follows: 8 (30.7%) were in the head and neck region (one conjunctiva, one oral cavity, one tongue, one ears, one head and three neck), 4 (15.3%) lumbar , 2 (7.7%) lumbosacral, 2 (7.7%) spinal site, 2 (7.7%) back region, 2 (7.7%) arms, 1 (3.9%) chest, 1 (3.9%) gluteus, 1 (3.9%) heel, 1 (3.9%) labium major, 1 (3.9%) axilla, and 1 (3.9%) fingers. Tumor types were fibrolipoma in three cases (11.5%), spindle cell lipoma in one case (3.9%), and intramuscular lipoma in two cases (7.7%)

While 24 of the cases presented to the clinic with complaints of swelling, 2 were detected incidentally.

The 11 cases with available radiological imaging findings showed a well-circumscribed mass lesion with soft tissue echogenicity.

Of the 19 lipoblastoma cases, 11 were male (57.9%) and 8 were female (42.1%). The mean age of these cases was 20.3+29.4 months (range: 1-108 months). The mean tumor diameter was 4.87+2.4 cm (range: 0.3-9 cm) with the exception of 2 cases with unavailable tumor diameter measurements as these cases were referred from other institutions for consultation.

The locations of lipoblastoma were as follows: the thigh in 4 cases (21.1%), gluteal area in 2 (10.6%), inguinal region in 2 (10.6%), feet in 2 (10.6%), back region in 2 (10.6%), neck in 1 (5.2%), supraclavicular region in 1 (5.2%), axilla in 1 (5.2%), left hand in 1 (5.2%), sacrum in 1 (5.2%), perineum in 1 (5.2%) and scrotum in 1 (5.2%).

All patients initially presented with complaints of swelling with the exception of 3 cases in which data on the cause of admission was unavailable.

**Figure 1:**
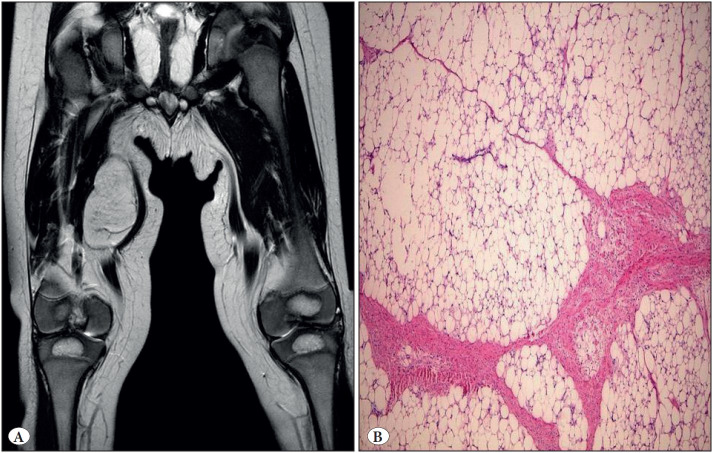
**A)** Radiographic image of T2A coronal section of lipoblastoma case. **B)** Lipoblastoma is composed of spindle shaped adipocytes admixed with multi-vacuolated or signet ring lipoblasts in the myxoid stroma (H&E; x100).

The radiological imaging findings were available in 10 cases, revealing a well-circumscribed, slightly heterogeneous and solid mass lesion with an echogenicity similar to adipose tissue (Figure 1).

Microscopically, there were single and multi-vacuolated adipocytes separated by fibrous septa, stellate-nodular cells and lobules composed of myxoid stroma. No nuclear atypia or mitosis was detected (Figure 1).

Of the lipoblastoma cases, 10 did not attend follow-up after the diagnosis while 9 had a follow-up period ranging from 3 to 72 months (mean: 26.1 months).

Two of the cases in our series of pediatric lipomatous tumors had atypical lipomatous tumor. One was an 8-year-old female who had a tumor localized in her left thigh with a diameter of 8 cm. The other case was a 10-year-old female who had a retroperitoneal tumor with a diameter of 17 cm. The radiological examination of these cases, who presented with complaints of swelling, revealed a mass lesion with an echogenicity similar to that of surrounding adipose tissue.

**Figure 2:**
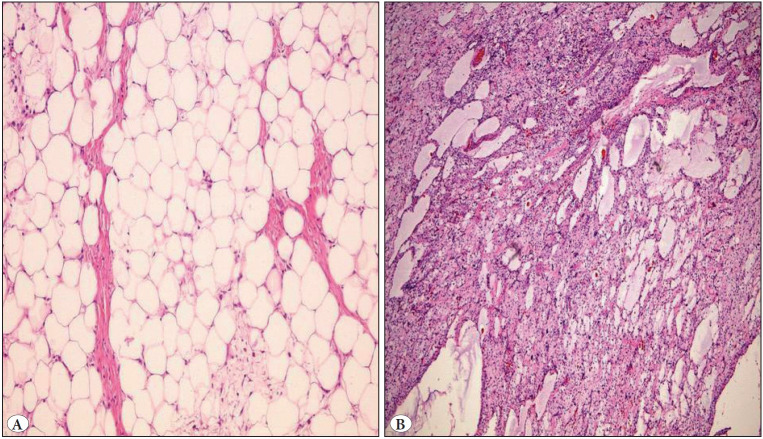
**A)** Well-differentiated liposarcoma composed of mature fat with variably sized adipocytes and bands of fibrotic stroma containing spindle cells with enlarged, hyperchromatic nuclei (H&E; x100). **B)** Myxoid liposarcoma composed of uniform, small spindle and oval cells and thin capillary network in the myxoid stroma (H&E; x100).

**Figure 3:**
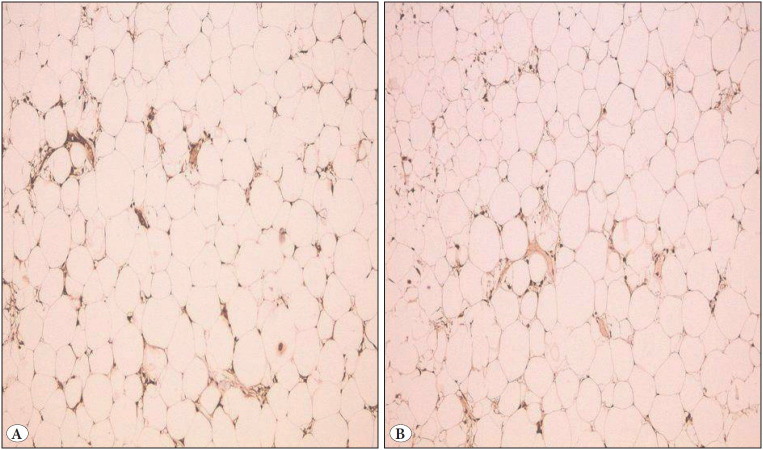
**A)** MDM2 positivity (IHC; x100). **B)** CDK4 positivity (IHC; x100).

Histopathological evaluation revealed hyperchromatic and large nucleus cells; lipoblasts were rarely more common in thin fibrous septa separating the adipocytes and there were differences in size and shape of the adipocytes. The tumor was separated from surrounding tissue with a thin fibrous capsule (Figure 2). The immunohistochemical analyses performed on the tumor of the first case were positive for MDM2 and CDK4 (Figure 3).

The pleomorphic liposarcoma case was a 15-year-old male with a 3 cm diameter tumor localized in his back region. We were unable to obtain the radiological findings of this case, who was admitted to the clinic with complaints of swelling.

Histopathological evaluation revealed a tumoral lesion composed of giant and pleomorphic spindle shaped cells with multivacuolar lipoblasts showing prominent pleomorphism. The surgical margins of the lesion were evaluated as intact. The immunohistochemical analyses performed on the tumor with total excision revealed negative staining for MDM2 and CDK4. Lipoblasts were stained positive for S100.

One of the myxoid liposarcoma cases was a 16-year-old female with a 10 cm diameter tumor localized in her right thigh. The other case was a 12-year-old female with a tumor of 4 cm diameter localized in her left popliteal fossa. We were unable to obtain the radiological findings of these cases, who were both admitted to the clinic with complaints of swelling and underwent total excision.

Histopathological evaluation revealed a tumoral lesion composed of uniform, small spindle and oval cells and thin capillary network in the myxoid stroma. There were extracellular mucin pools in the tumor. A fibrous pseudocapsular structure surrounded the multinodular tumor and the surgical margins were evaluated as intact (Figure 2). The second myxoid liposarcoma case developed recurrence two years later. All of the liposarcoma cases are still alive and healthy.

## DISCUSSION

Lipomatous tumors comprise approximately 6% of all soft-tissue tumors in children (8). Data on the prevalence and incidence of these tumors is insufficient since there is a limited number of studies on pediatric lipomatous tumors in the literature. Benign lipomatous tumors accounted for 8.73% of the total of 385 cases in the pediatric tumor series of Punia et al. in which 11 of the 24 cases were between 0 and 4 years of age, and the female to male ratio was 0.84 (9). In our study, this ratio was 0.92.

Lipoma is a benign mesenchymal tumor composed of mature lipocytes. It is especially common in adults aged 40 - 60 years and rarely occurs before 20 years of age (1,10,11).

The definite etiology of lipoma has not yet been clarified, although publications suggest that genetic, endocrine, and traumatic factors play a role. Lipoma may also accompany various syndromes such as Gardner’s syndrome, Madelung’s disease, and Dercum’s disease (12).

Lipomas can be subcutaneous or deep-seated. Deep-seated lipomas are divided into two types as intramuscular (situated between muscle fibers) and intermuscular (admixed with muscle fibers). Intramuscular lipomas are typically localized in the chest wall, head-neck region, and lower and upper extremities, while intermuscular lipomas are located in the anterior wall of the abdomen. Retroperitoneal lipomas, however, are very rare. Periosteal lipoma (bone surface-seated) and lipoma arborescence (synovium-seated) are lipomas specifically named according to their locations (1,5,13,14). In our series, 2 of the 24 cases had intramuscular and 2 had intradural extramedullary lipomas, with the remaining cases being of the superficial type.

The cases had clinical presentations as mobile, painless, and palpable masses; however, due to their slow-growing nature, lipomas can also be detected incidentally without any symptoms. Larger lipomas may also cause various symptoms by compressing surrounding tissue (15). In our series, the most common cause of admission was complaints of swelling according tuo the data obtained from patient records.

Ultrasonography is the preferred method of radiological imaging for lipomas. The general ultrasonographic appearance is hyperechoic, admixed with the surrounding muscle tissue and parallel to the skin, as an elliptical or rounded mass lesion . MR or CT imaging may be helpful in assessing the lesion and margins appear as fuzzy-bordered or large-sized masses (16). In the 11 cases with accessible radiological imaging in our series, the ultrasonographic findings were in the form of a well-defined mass lesion with an echogenicity of soft tissue.

Although typically smaller than 5 cm, the literature mentions “giant lipomas” larger than 10 cm, weighing over 1000 gr (2,11). Our lipoma cases were macroscopically well-defined, homogeneous mass lesions with yellow cross sections, separated from the surrounding tissue with thin fibrous capsules. The average diameter in our series was 4.84±3.1 cm.

Microscopically, lipomas appear as well-defined masses composed of mature adipocyte lobules. As an exception, intramuscular lipomas have borders that appear infiltrative. Variants include angiolipoma, spindle cell lipoma, pleomorphic lipoma, chondroid lipoma, myolipoma, myelolipoma, fibrolipoma, and fibrohistiocytic lipoma. Well-differentiated liposarcoma is especially important in the differential diagnosis of intramuscular tumors. The features that distinguish lipoma from liposarcoma include pleomorphism, mitosis, hyperchromatic nucleus, and absence of bizarre cells and lipoblasts (1,2,17). In our series of 24 cases, 3 were fibrolipoma, 2 were intramuscular lipoma, and the remaining cases had typical lipoma morphology.

Molecular genetic examinations of the lipomas revealed ring/giant chromosomes and the rearrangement of the HMGA2 gene (1,18). In the lipoma tumor series by Bartuma et al., 8 of the 272 cases were between 0 and 20 years of age and 3 of these 8 cases had rearrangement of 12q13, one had rearrangement of 6p21, one had a ring chromosome, and 3 had different karyotypic abnormalities (19).

Lipoblastoma is a benign mesenchymal tumor arising from embryonic white fat tissue. These tumors, which rarely accur in adolescents and adults, account for less than 1% of all childhood tumors (20).

Lipoblastomas are more common in males than females, and 90% of them are diagnosed before 3 years of age (1,21). In our series, the mean age of the cases was 20.3±29.4 months with a male predominance as consistent with the literature.

Lipoblastomas can be divided into two groups according to their locations and margins with the surrounding tissue. The term lipoblastoma refers to superficial, encapsulated, and well-defined tumors, whereas the term lipoblastomatosis refers to deep-seated infiltrative tumors (22). All cases in our series were superficial tumors with lipoblastoma morphology.

Lipoblastomas are most commonly located in the trunk and extremities, but may also occur in other regions including the retroperitoneum, pelvis, abdomen, mesentery, mediastinum, head and neck, and solid organs (23). In their series of 32 cases, Speer et al. reported that 12 of the cases were located in the trunk, 12 were in the extremities, 5 were in the inguinal region and 3 were in the neck (24). In our series, the locations of tumors included the extremities in 7, the gluteal area in 2, the inguinal region in 2, the back region in 2, the supraclavicular area in 1, the axillary area in 1, the sacrum in 1, the perineum in 1, and the scrotum in 1.

Similar to lipomas, if any symptoms occurred, the clinical presentations of the cases were with complaints of mobile, painless and palpable masses. When they attain large sizes, pressure on the surrounding tissues may cause various symptoms (25). According to the data obtained from archive reports, the most common complaint at presentation was swelling in our series.

Although radiological imaging is considered useful in diagnosing lipoblastoma, a radiological finding by itself does not rule out malignancy. MRI is more preferred in lipoblastomas than CT, with higher signal intensity and heterogeneity in T1-weighted images, and moderate in T2-weighted images (26,27). The 10 cases with available ultrasonography reports in our series, showed a well-defined, mildly heterogeneous, solid mass lesion with an echogenicity similar to adipose tissue.

These tumors were macroscopically well-defined, lobulated, soft mass lesions with yellow-white cross sections. There were also myxoid areas in some tumors. They were typically 2-5 cm in size, but they can also be larger (1,2). In our series, the mean tumor diameter was 4.87+2.4 cm (range: 0,3-9 cm).

Microscopically, there were single and multi-vacuolated adipocytes separated by fibrous septa, stellate-nodular cells and lobules composed of myxoid stroma. The findings of nuclear atypia and mitosis were not anticipated (28). Microscopic findings were consistent with the literature in our series.

Molecular genetic examinations of lipoblastomas revealed rearrangements of 8q11∼q13, hyaluronic acid synthase 2 (HAS2) and collagen 1 alpha 2 (COL1A2) as well as pleomorphic adenoma gene 1 (PLAG1) amplification (29).

Liposarcomas are malignant tumors with differentiated adipose tissue. These tumors are common in adults; however, they account for less than 3% of pediatric soft-tissue sarcomas (30). There are publications reporting a mild female predominance in these tumors which are more common particularly in the second decade in pediatric patients. Ferrari et al. reported the average age of diagnosis as 7 years in their series of 12 cases. The female to male ratio of the cases was 0.5 (31). In our series, the average age of diagnosis as 12.2 in 5 liposarcoma cases (range: 8-16).

Although liposarcomas are mostly localized in the extremities, they can also occur in pelvis, inguinal region, head and neck, chest cavity, axilla, mediastinum, visceral space such as abdominal cavity, and retroperitoneum. Well-differentiated and pleomorphic types of liposarcoma are mostly located in the retroperitoneum and visceral cavity. Studies have shown that tumor localization is associated with histological subtype (7). In their pediatric liposarcoma series of 17 cases, Shmookler et al. reported that the tumors were mostly localized in the lower extremity in 5 cases (32). Of the 5 liposarcoma cases in our series, diagnosed as well-differentiated liposarcomas, localization was in the lower extremity and retroperitoneum; the myxoid liposarcomas were localized in the lower extremity while the single case of pleomorphic liposarcoma was localized in the back region.

The most common type of liposarcoma in the pediatric age group is myxoid/round-cell liposarcoma, which consists of well-differentiated, de-differentiated, myxoid/round-cell and pleomorphic subtypes. Approximately 5% of liposarcomas do not carry the characteristics of a particular subtype, and are therefore called “mixed type liposarcoma”. Liposarcomas in adults have macroscopic and microscopic features that are similar to those in the pediatric age group. They are generally large-sized (> 5cm), multinodular, pale yellow or bronze colored masses. They may include regions of hemorrhage and necrosis as well as myxoid-gelatinous or non-lipogenic areas depending on the type of liposarcoma. Unlike benign lipomatous tumors, liposarcomas may have infiltrative borders, and are composed of lipoblasts, thick fibrous septa, hyperchromatic atypical cells and atypical mitosis (1,2,30,33). In their series of 82 cases under 22 years of age, Allagio et al. found that 58 cases (71%) were diagnosed with myxoid and round-cell liposarcoma, 12 cases (15%) with pleomorphic myxoid liposarcoma, 6 cases (7%) with spindle cell myxoid liposarcoma, 4 cases (5%) with well-differentiated liposarcoma, and 2 cases (2%) with pleomorphic liposarcoma (34). In our series, 2 cases (4%) were diagnosed with atypical lipomatous tumor, 2 cases (4%) with myxoid liposarcoma and 1 case (2%) with pleomorphic liposarcoma.

Molecular and cytogenetic examination of liposarcomas revealed ring chromosomes and long determinant chromosomes originated from the 12q13-15 region, amplifications of MDM2, CDK4 and HMGA2 in well-differentiated and dedifferentiated liposarcomas as well as translocations of (12;16)(q13;11) and (12;22)(q13;q22) caused by rearrangements of FUS-CHOP or EWSR1-CHOP in myxoid liposarcomas. There were complex karyotypic abnormalities in pleomorphic liposarcomas but no specific cytogenetic abnormality was found (35).

In addition to assessment of morphological features, immunohistological staining is an adjuvant method in distinguishing between malignant and benign lipomatous tumors. MDM2 and CDK4 nuclear staining is not a characteristic of benign lipomatous tumors in well-differentiated and dedifferentiated liposarcomas; however, it should be noted that it is not encountered in myxoid and round cell pleomorphic liposarcomas (2,36). In our series, the immunohistochemical analyses performed on the first case of atypical lipomatous tumor stained positive for MDM2 and CDK4.

In the differential diagnosis of liposarcoma, Ewing/PNET group sarcomas, rhabdomyosarcoma, neuroblastoma, extraskeletal mesenchymal chondrosarcoma have an important place as the myxoid-round cell liposarcoma is the most common subtype in pediatric age group (2). Immunohistochemical and molecular findings as well as histomorphological findings are also useful. Detailed information on benign and malignant entities in the differential diagnosis is presented in Table 2.

**Table 2:** Major entities in differential diagnosis of liposarcomas.

**Benign entities**			
	**Clinical features**	**Microscopic features**	**Assistive techniques**
Fat necrosis	There is no specific feature. The history of trauma is significant in the anamnesis.	Necrotic lipomatous tissue, foamy macrophages Lipoblast-like cells may be seen	While MDM2 and CDK4 are generally negative, macrophages show positive staining with CD68.
Lipoma containing fat necrosis	Generally good limited, superficial masses. The anamnesis may include a history of trauma.	Mature adipocytes and fat necrosis findings without hyperchromatic-large cells	While MDM2 and CDK4 are generally negative, macrophages show positive staining with CD68. HMGA2 gene variations are detected.
Silicone granuloma	They develop due to the rupture of the implant in patients with a history of mammary implant.	A number of lipoblast-like cells and inflammatory reactions are detected between the lobules and the ductus.	While MDM2 and CDK4 are generally negative, macrophages show positive staining with CD68.
Spindle cell lipomas	Superficial masses typically located at the shoulder and back.	Lipomatous areas with non-atypical collagenized spindle cells are observed.	While MDM2 and CDK4 are generally negative, CD34 is positive. Unbalanced karyotypes / hypodiploidy, partial losses and monosomy 13 are detected.
Pleomorphic lipomas	They are superficial masses typically located at the shoulder and back.	Lipomatous areas with non-atypical collagenized spindle cells and floret-like hyperchromatic giant cells.	While MDM2 and CDK4 are generally negative, CD34 is positive. Unbalanced karyotypes / hypodiploidy, partial losses and monosomy 13 are detected.
Hibernomas	Generally located at the thigh at an early age.	A lobulated lesion lacking hyperchromasia which consists of intracytoplasmic vacuolar adipocytes.	Chromosome 11q13 abnormalities are detected.
Myxomas	Generally located at the thigh, shoulder and upper arm in adults.	Hypocellular lesions with poor vascular structures.	While spindle cells are positive with CD34 and Vimentin staining, they are generally negative with S100 and Desmin staining. GNAS mutation is present.
**Malignant Entities**			
Undifferentiated pleomorphic sarcoma	Usually detected in adult patients as deeply localized.	They are tumors consisting of pleomorphic spindle cells with storiform pattern.	While MDM2 and CDK4 are generally negative, variable SMA and Desmin positivity may be detected.
Ewing/PNET group sarcomas	Generally young.	They are tumoral lesions with round or oval shape with vesicular nucleus, fine chromatin, narrow cytoplasm and uniform appearance.	While CD99 and FLI-1 are positive, neuroendocrine markers can detect positivity, too. EWSR1 translocation is detected.
Rhabdomyosarcomas	Generally young.	They are spindle cell tumors with no lipoblasts, containing cells with significant eosinophilic cytoplasm.	Desmin, MyoD1 and Myogenin are detected positive. FOXO1 translocation is observed in alveolar type rhabdomyosarcoma.
Neuroblastomas	Generally under 2 years of age.	They are tumors consisting of round or oval shaped cells with minimally amphophilic cytoplasm and varying proportions of neurofibrillary matrix.	Positive staining is detected by neuroendocrine markers. nMYC amplification can be seen.
Myxofibrosarcomas	Superficial masses spreading throughout the fascia in patients with advanced age.	They are myxoid tumors rich in pleomorphic spindle cells and thick walled vessels.	Generally CD34 and SMA positivity are detected. However, there is no specific immunuhistochemical staining.
Other sarcomas infiltrating the surrounding fat tissue		Atypia, shape-size differences are only detected in adipocytes located in the surrounding fat tissue.	MDM2 and CDK4 are negative.

Treatment of benign and malignant lipomatous tumors involves a total excision with safe margins. Well-differentiated and low-grade tumors generally have better prognosis and do not develop any distant metastasis. These tumors should be investigated for the possibility of local recurrence.

Studies report that total excision and radiotherapy should be performed together to prevent local recurrence. Poorly differentiated and high-grade tumors have poor prognosis and may establish distant metastases. In addition to liposarcomas, 10-20% of recurrence can be detected in lipoblastomatosis, particularly in lipoblastomas (2,33,37,38). We found no recurrence in the 9 lipoblastoma cases in the follow-up of our series. We found recurrence in one of the myxoid liposarcoma cases after two years. However, the other cases of liposarcoma could not be evaluated in terms of recurrence or metastasis.

In conclusion, lipomatous tumors are the most common type of mesenchymal tumors in adults, but rarely occur in children. Tumors localized in deep tissues may especially slowly enlarge to greater sizes and cause various clinical symptoms by compressing surrounding tissues. Local recurrences, which may develop even after total excision, necessitate close monitoring of the cases.

## Conflict of Interest

The authors declare no conflict of interest.
